# Extracts from Six Native Plants of the Yucatán Peninsula Hinder Mycelial Growth of *Fusarium equiseti* and *F. oxysporum*, Pathogens of *Capsicum chinense*

**DOI:** 10.3390/pathogens9100827

**Published:** 2020-10-10

**Authors:** Patricia Cruz-Cerino, Jairo Cristóbal-Alejo, Violeta Ruiz-Carrera, Germán Carnevali, Marina Vera-Ku, Jesús Martín, Fernando Reyes, Marcela Gamboa-Angulo

**Affiliations:** 1 Unidad de Biotecnología, Centro de Investigación Científica de Yucatán, 97205 Mérida, Mexico; 2 Laboratorio de Fitopatología, Tecnológico Nacional de México, Instituto Tecnológico de Conkal, 97345 Conkal, Mexico; jairo.cristobal@itconkal.edu.mx; 3 Laboratorio de Biotecnología, Universidad Juárez Autónoma de Tabasco, 86039 Villahermosa, Mexico; violeta@ujat.mx; 4 Unidad de Recursos Naturales, Centro de Investigación Científica de Yucatán, Chuburná de Hidalgo, 97205 Mérida, Mexico; carneval@cicy.mx (G.C.); marina.vera@cicy.mx (M.V.-K.); 5 Orchid Herbarium of Oakes Ames, Harvard University Herbaria, Cambridge, MA 02138, USA; 6 Fundación MEDINA, 18016 Granada, Spain; jesus.martin@medinaandalucia.es (J.M.); fernando.reyes@medinaandalucia.es (F.R.)

**Keywords:** antifungal, α-asarone, habanero pepper, phytopathogens, *Mosannona depressa*, plant extracts

## Abstract

*Fusarium equiseti* strain FCHE and *Fusarium oxysporum* strain FCHJ were isolated from the roots of wilting habanero pepper (*Capsicum chinense* Jacq.) seedlings with root rot. Toward developing a biorational control of these serious phytopathogenic strains, ethanolic (EE) and aqueous (AE) extracts of different vegetative parts of 40 tropical native plants of the Yucatán Peninsula were screened for antifungal activity. Extracts of six out of 40 assayed plants were effective, and the most inhibitory extracts were studied further. EEs from *Mosannona depressa* (bark from stems and roots), *Parathesis cubana* (roots), and *Piper neesianum* (leaves) inhibited mycelial growth of both strains. Each active EE was then partitioned between hexane and acetonitrile. The acetonitrile fraction from *M. depressa* stem bark (MDT-b) had the lowest minimum inhibitory concentration of 1000 µg/mL against both pathogens and moderate inhibitory concentration (IC_50_) of 462 against *F. equiseti* and 472 µg/mL against *F. oxysporum*. After 96 h treatment with EE from *M. depressa* stem bark, both strains had distorted hyphae and conidia and collapsed conidia in scanning electron micrographs. Liquid chromatography–ultraviolet–high resolution mass spectrometry analysis revealed that the major component of the fraction was α-asarone. Its antifungal effect was verified using a commercial standard, which had an IC_50_ of 236 µg/mL against *F. equiseti* and >500 µg/mL against *F. oxysporum*. Furthermore, the *P. cubana* hexane fraction and *P. neesianum* acetonitrile fraction had antifungal activity against both *Fusarium* pathogens. These compounds provide new options for biorational products to control phytopathogenic fungi.

## 1. Introduction

Approximately 200 species of *Fusarium* are recognized as pathogens of a broad range of plants, and *F. graminearum* and *F. oxysporum* were ranked in fourth and fifth place among the top 10 scientifically or economically most important fungal pathogens [1]. In pepper (*Capsicum* spp.) crops, serious post-harvest losses are caused by *F. oxysporum* [2]. Peppers from about 35 *Capsicum* species are consumed, most widely from *C. annuum*, *C. baccatum*, *C. frutescens*, *C. pubescens* and *C. chinense,* which have been the most successfully domesticated and cultivated [3]. México reported an annual production of 3.2 million tons of pepper crops and average annual growth in production of 4.82% during 2003–2016 [4]. In particular, habanero peppers (*C. chinense*) are appreciated worldwide for their high content of capsaicin, the main alkaloid responsible for their hotness [5]. Capsaicin is also beneficial as a cardioprotective, anti-inflammatory, analgesic and a gastrointestinal aid and for its thermogenic properties [6]. In the chemical industry, it is useful in the production of paints and varnishes, tear gas and other compounds. In the Yucatán Peninsula, habanero peppers are part of the culinary identity as a condiment [7]. The habanero pepper from the Yucatán Peninsula Denomination of Origin (NOM-189-SCFI-2017) is presently cultivated on 1134 ha [8], and its production has been increasing steadily in recent years. However, *Fusarium* spp. cause production losses of at least 50% or even 100% when conditions are favorable [9]. *F. oxysporum* and *F. equiseti*, which infect the roots of habanero pepper seedlings and cause root rot and wilting in the Yucatán Peninsula, México [10], also produce mycotoxins such as fumonisins and trichothecenes in crops and feed products and represent a risk to human health [11].

Currently, the management of *Fusarium* species depends on the intensive use of synthetic fungicides such as a benomyl, carbendazim, thiabendazole and alliete [12]. However, such intensive use can induce resistance in the pathogen and negatively impact the environment, beneficial microorganisms and humans by acting as a skin irritant and carcinogen [13,14]. To reduce dependence on synthetic pesticides, numerous strategies, such as the rotation of crops, use of resistant cultivars and biorational products and solarization of the soils, are thus integrated into a pest management program [15,16]. Natural products derived from plants are a highly viable option as biorational antifungal products that should leave less environmental residue and be nontoxic to beneficial organisms and humans [17,18]

To discover and incorporate new antifungal agents in the control of diseases caused by *Fusarium* species, several groups have tested plant extracts in vitro and in vivo [19,20,21]. The high plant diversity in Mexico, with 23,314 reported species, 50% of which are endemic, has scarcely been explored for their biological and chemical properties. In the Yucatán Peninsula, the 2330 known species of vascular plants, belonging to 956 genera and 161 families, represent 6% of the Mexican flora [22,23]. Previous bioprospecting of Yucatecan native plant extracts for activity against phytopathogenic fungi has revealed good fungicidal properties of extracts from plants such as *Acacia pennatula*, *Acalypha gaumeri* and *Croton chichenensis* [24,25,26].

Because of the increasing demand for natural fungicides to control habanero pepper diseases, more bioprospecting programs have been needed. Therefore, here, we screened 184 extracts from 40 plant species native to the Yucatán Peninsula for activity against *F. equiseti* FCHE and *F. oxysporum* FCHJ strains from habanero pepper (Table 1), examined hyphae using scanning electron microscopy (SEM) for any morphological effects of the active extracts and analyzed the chemical profile of the active fractions obtained from active extracts using liquid chromatography–ultraviolet–high-resolution mass spectrometry (LC-UV-HRMS).

## 2. Results

### 2.1. Antifungal Activity of Plant Extracts Against Fusarium spp.

Table 2 shows the results of active plant extracts on mycelial growth of *F. equiseti* FCHE and *F. oxysporum* FCHJ. Ethanolic extracts (EEs) from *Mosannona depressa* (bark of stem and root), *Parathesis cubana* (root) and *Piper neesianum* (leaves) at 2000 µg/mL and aqueous extracts (AE) from *Cameraria latifolia* (root), *Calea jamaicensis* (whole plant) and *Heteropterys laurifolia* (leaves) at 3% *w*/*v* were active against one or both *Fusarium* strains after 96 h. All these active extracts inhibited mycelial growth of *F. equiseti*, but only four EEs inhibited mycelial growth of *F. oxysporum*. No active AEs were detected against *F. oxysporum*.

Complete mycelial growth inhibition (MGI of 100%) for both phytopathogens was achieved with EEs from *M. depressa* bark of stems and *P. cubana* roots. The EE from leaves of *P. neesianum* was also effective (MGI of 100% against *F. equiseti* and 75% against *F. oxysporum*). The AE from *C. jamaicensis* also achieved 75% MGI against *F. equiseti*. The EAs from *C. latifolia* root and *H. laurifolia* leaves achieved MGI of only 25% against *F. equiseti* (Table 2). On the other hand, none of the EAs had any activity against *F. oxysporum*. The positive control, prochloraz (0.11%), completely inhibited the growth of the two phytopathogens, and typical mycelial growth of both plant pathogens was observed for the negative controls. The other plant extracts did not cause significant mycelial growth inhibition with respect to the negative control (Supplementary Table S1).

### 2.2. Minimum Inhibitory Concentration of Ethanolic Extracts, Fractions and α-Asarone

The minimum inhibitory concentration (MIC) of the four EEs that completely inhibited mycelial growth of both *Fusarium* strains was determined. *F. equiseti* was more sensitive to the extracts from *M. depressa* stem bark, *P. cubana* roots and *P. neesianum* leaves (MIC: 1000 µg/mL). All these active extracts were fungicidal, except for the extract from leaves of *P. neesianum*, which was fungistatic (Table 2). In contrast, *F. oxysporum* was less sensitive to the four EEs, which were fungistatic and had MICs of 2000 µg/mL. Therefore, the four EEs were partition-fractionated, and serial dilutions of each fraction (hexane, acetonitrile and a methanol-soluble precipitate) were tested for activity.

The most active fractions against *F. oxysporum* were the hexane (MDT-a) and acetonitrile (MDT-b) fractions from *M. depressa* stem bark, which were both fungistatic, and the hexane fraction from *P. cubana* roots (PCR-a), which was fungicidal; all had a MIC of 1000 µg/mL (Table 3). As expected, a fungicidal effect on *F. equiseti* was induced by half of the fractions, with a MIC of 1000 µg/mL. These fractions were the same as those that inhibited *F. oxysporum*: the acetonitrile fraction from *P. neesianum* leaves (PNH-b), precipitates of *P. cubana* roots (PCR-c) and *P. neesianum* leaves (PNH-c). The fractions obtained from the root bark and the precipitate of the stem bark of *M. depressa* were considered as inactive against the two pathogens because their MIC was greater than 1000 µg/mL (Table 3).

The MIC of the commercial α-asarone standard, evaluated in parallel with the fractions, was 500 µg/mL against *F. equiseti* with fungistatic effect, and >500 µg/mL against *F. oxysporum* (Table 3).

### 2.3. Inhibitory Concentration (IC_50_ and IC_95_)

The α-asarone standard had the lowest IC_50_ and IC_95_ against both species, followed by the MDT-b fraction from *M. depressa* stem bark (Table 4). Interestingly, the IC_50_ and IC_95_ for the MDT-b fraction and α-asarone were very similar against *F. oxysporum* (respectively, 472 and 539 µg/mL, MDT-b, 482 and 526 µg/mL, α-asarone). Against *F. equiseti*, the IC_50_ and IC_95_ for the MDT-b and PNH-b fractions were both 462 and 526 µg/mL, respectively, higher than for α-asarone and similar to those for the EE from *M. depressa* stem bark.

### 2.4. Effect of Active Extracts from Mosannona depressa on Morphology of Fusarium Strains

**Figure 1:**
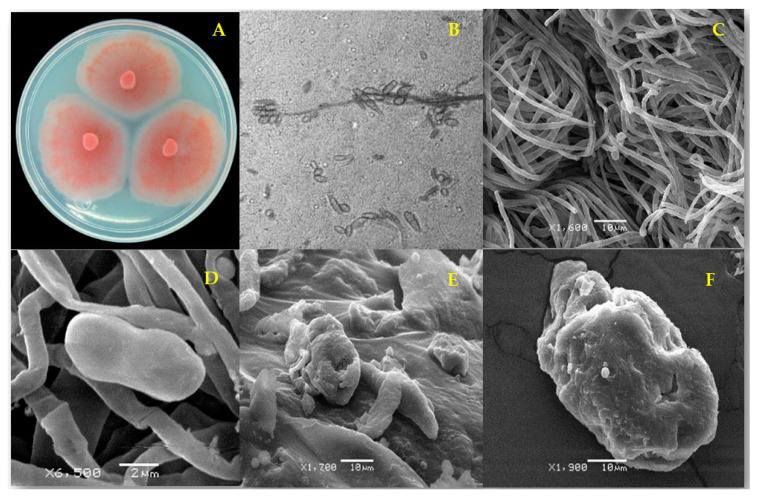
*Fusarium equiseti* strain FCHE morphology (**A**) after 7 d on potato dextrose agar; (**B**) microconidia of *F. equiseti* (1000×) and (**C**) typical untreated mycelium and microconidia (negative control); (**D**) apparently normal microconidium and (**E**) distorted mycelium and collapsed microconidia after 96 h treatment with ethanolic extract from *Mosannona depressa* stem bark at 2000 µg/mL; (**F**) rough surface of a collapsed-looking microconidium after 96 h treatment with 2000 µg/mL ethanolic extract from *M. depressa* root bark.

**Figure 2:**
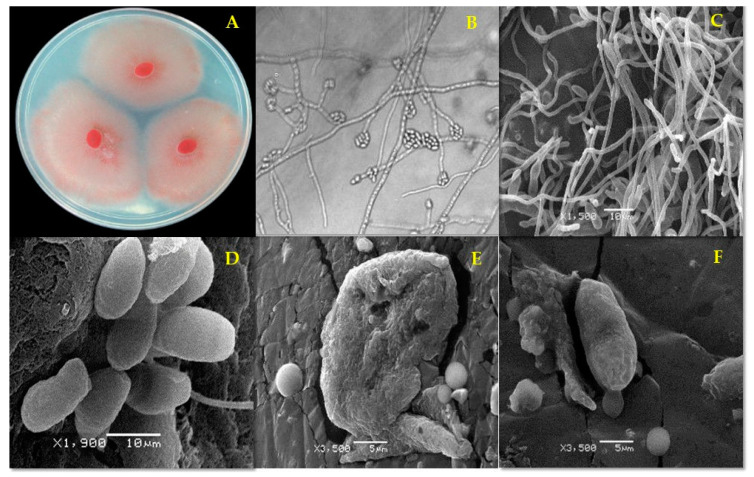
*Fusarium oxysporum* strain FCHJ morphology (**A**) after 7 d on potato dextrose agar (PDA). (**B**) Microconidia (1000×) and (**C**) typical mycelium and microconidia (negative control); (**D**) apparently normal microconidia, (**E**) misshapen and collapsed microconidium after 96 h treatment with ethanolic extract from *Mosannona depressa* stem bark at 2000 µg/mL; (**F**) collapsed conidium after 96 h treatment with ethanolic extract from *M. depressa* root bark at 2000 µg/mL.

The SEM of the untreated strains (negative control) showed typical well-formed hyphae and microconidia (Figure 1A–D and Figure 2A–D). After 96 h of exposure to 2000 µg/mL EE from *M. depressa* stem bark, *F. equiseti* had distorted hyphae, globular structures along the surface of the mycelium and contorted and dehydrated conidia (Figure 1E). Conidia of the same strain were similarly affected by 2000 µg/mL EE from *M. depressa* root bark (Figure 1F).

Exposure of *F. oxysporum* to EE from *M. depressa* stem bark at 2000 µg/mL also led to malformed hyphae and contorted, dehydrated microconidia (Figure 2E), while EE from *M. depressa* root bark at 2000 µg/mL induced dehydration and distortion of hyphae and dehydration of microconidia (Figure 2F).

### 2.5. Identification of Active Components in Extracts from Mosannona depressa by LC-UV-HRMS

**Figure 3:**
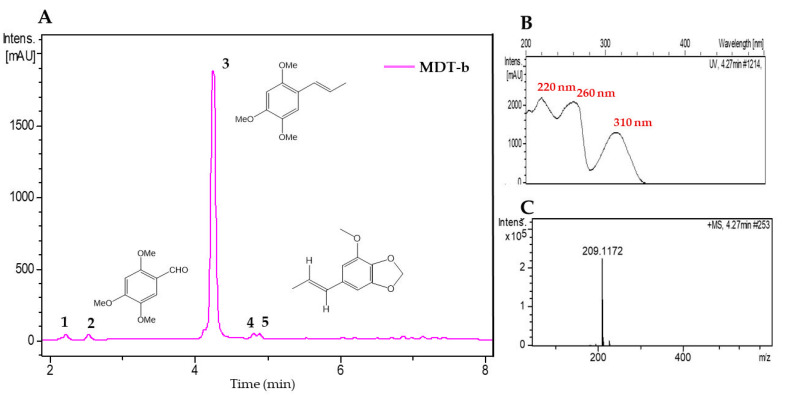
**(A**) Liquid chromatogram (UV 210 nm) of acetonitrile fraction from stem bark of *Mosannona depressa* (MDT-b). **1**: Not identified (C_12_H_16_O_4_), **2**: asaraldehyde, **3**: α-asarone, **4**: not identified, **5**: *trans*-isomyristicin. (**B**) UV spectrum of peak **3** and (**C**) high-resolution mass spectrum of peak **3**.

The MDT-b and MDR-b fractions from *M. depressa* bark from the stem and roots were analyzed by LC-UV-HRMS (Table 5). The chromatogram of the MDT-b fraction showed five components, with the most abundant eluted at a retention time of 4.27 min (peak 3, Figure 3). The HRMS of peak 3 presented a protonated molecular ion at *m/z* 209.1172, indicative of a molecular formula of C_12_H_16_O_3_ (calc. for C_12_H_17_O_3_^+^, 209.1173), and its UV spectrum exhibited maxima at 220, 260 and 320 nm. This component was identified as α-asarone based on the reference spectrum in the equipment databases and confirmed using a commercial standard (Figure 3, Table 5). The minor components at retention times of 2.33, 2.55, 4.8 and 4.89 min had structural characteristics similar to those of α-asarone, and their UV and HRMS data were compared with databases in the literature and Chapman & Hall Dictionary of Natural Products (CHDNP). The HRMS of peak 1 showed UV maxima at 230 and 290 nm, and a protonated ion at *m/z* 225.1120, suggesting a molecular formula of C_12_H_16_O_4_ (calc. for C_12_H_17_O_4_^+^, 225.1121), which was not assigned to any previously reported compound after comparison of the UV and HRMS data with databases in the literature and CHDNP. The analysis of peak 2 showed a protonated ion at *m/z* of 197.0808, with a molecular formula of C_10_H_12_O_4_ (calc. for C_10_H_13_O_4_^+^, 197.0808) and UV maxima at 238, 270 and 345 nm; thus, the compound was tentatively identified as asaraldehyde. Components 4 and 5 had the same UV maxima at 220, 240 and 290 nm and protonated ions at *m/z* 221.1170 and 193.0857, respectively, accounting for molecular formulae of C_13_H_16_O_3_ (calc. for C_13_H_17_O_3_^+^, 221.1172) for component 4 and C_11_H_12_O_3_ (calc. for C_11_H_13_O_3_^+^, 193.0859) for component 5. After an exhaustive comparison of their spectral data with CHDPN and databases in the literature, compound 5 was tentatively identified as isomyristicin, but compound 4 was not identified (Table 5).

**Figure 4:**
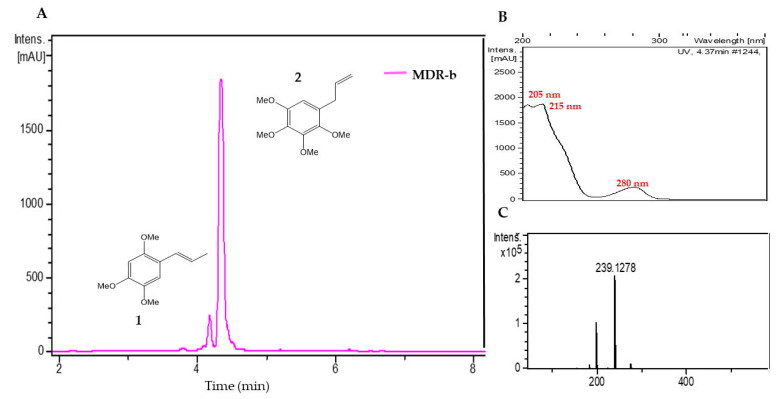
**(A**) Liquid chromatogram (UV at 210 nm) of the acetonitrile fraction from *Mosannona depressa* root bark (MDR-b); **1**: α-asarone **2**: 1,2,3,4-tetramethoxy-5-(2-propenyl) benzene. (**B**) UV spectrum of peak 2 and (**C**) high-resolution mass spectra of peak **2**.

Two components were detected in the medium polarity fraction (MDR-b) from *M. depressa* root bark (Figure 4). The most abundant was peak 2, with a retention time of 4.37 min, showing a protonated ion at *m/z* 239.1278, with a molecular formula of C_13_H_18_O_4_ (calc. for C_13_H_19_O_4_^+^, 239.1278); its UV spectrum presented maxima at 205, 215 and 280 nm. Comparison of these data with the databases led us to tentatively identify peak 2 as 1,2,3,4-tetramethoxy-5-(2-propenyl) benzene (Table 6). Data for peak 1 at a retention time of 4.25 min corresponded to α-asarone (Table 6).

## 3. Discussion

This first bioprospecting report on plant extracts with activity against fungal pathogens of habanero pepper is part of continuing efforts to discover potential bioactive compounds in the diverse flora of southeastern México. From sites not previously explored, we collected 40 plant species that our exhaustive search of the literature showed had not been tested against fungal phytopathogens, with the exception of *Annona primigenia* [28,29] and *Mosannona depressa* [30,31]. Antifungal screening of EEs and AEs from different vegetative parts of the 40 plant species led to the detection of six (15% of the total) species with activity against the *Fusarium* strains tested. These active extracts were from *Calea jamaicensis*, *Cameraria latifolia*, *Heteropterys laurifolia, Mosannona depressa*, *Parathesis cubana* and *Piper neesianum*. Interestingly, these plant species belong to different families and were collected at the same site, Jahuactal, a tropical evergreen rainforest with trees exceeding 20 m in height.

*Fusarium equiseti* was more sensitive than *F. oxysporum* to the plant extracts tested. Mycelial growth of *F. oxysporum* was inhibited by only four EEs, representing 4.3% of the plant extracts tested, and totally insensitive to AEs at the tested concentration (3% *w*/*v*). Several studies have indicated that AEs, even at higher concentrations, have limited effect on *F. oxysporum*. For example, the mycelial growth of *F. oxysporum* was inhibited 10–55% by extracts from leaves at 10% *w*/*v* of *Ocimum sanctum* [32] and stems, root and fruits of *Momordica charantia* [33], among others.

In contrast, in our study, four EEs completely inhibited the mycelial growth of both plant pathogens; the EEs from the bark of stems and roots of *M. depressa* were especially effective. Native to Mexico and Central America, this medicinal tree (syn. *Annona depressa*, *Guatteria gaumeri, Malmea depressa* and *M. gaumeri*) has a wide range of biological activities in humans, e.g., antifungal, antiproliferative, antiprotozoal, cytotoxic, hypoglycemic and hypocholesterolemic [34,35,36]. For agriculture applications, however, a chloroform extract from the stem bark of *M. depressa* was reported only as a growth inhibitor of *Amaranthus hypochondriacus* (IC_50_ = 134 µg/mL) and *Echinochloa crusgalli* (IC_50_ = 457 µg/mL), and as a fungicide against *F. oxysporum* (MIC = 400 µg/mL) [31]; EEs from *M. depressa* stem and root bark had antifungal activity against *Penicillium oxalicum* (MIC = 250 µg/mL) [37].

The present report is also the first on the fungicidal effect of the EEs from *M. depressa* against *F. equiseti*. The MIC of 1000 µg/mL for EEs from the bark of stems and roots of *M. depressa* is comparable to the effect against *F. equiseti* reported for ethanolic extracts of leaves from *Calycopteris floribunda* (MIC: 500 µg/mL) [38] and rhizomes from *Acorus calamus* (MIC: 1000 µg/mL) [39]. In the case of *F. oxysporum*, here, both EEs from the bark of stems and roots of *M. depressa* were fungistatic with a higher MIC of 2000 µg/mL. In a previous study, a chloroform extract of the stem bark of *M. depressa* was antifungal against *F. oxysporum* (MIC: 400 µg/mL) and *Trichophyton mentagrophytes* (MIC: 500 µg/mL) [31]. The lower MIC may be attributed to the polarity of the solvent used and the susceptibility and forma specialis of the pathogenic strain tested [40]. Matos et al. [41] found variation in the sensitivity to *Chelidonium majus* extracts among six *F. oxysporum* isolates, with f. sp. *cubense* the most sensitive.

The guided fractionation with the antifungal assay of the EEs from the bark of stems and roots of *M. depressa* showed that *F. equiseti* and *F. oxysporum* were more sensitive to the MDT-b fraction. LC-UV-HRMS analyses revealed a mixture of phenylpropanoids in the MDT-b fraction; the major component was α-asarone, with minor components asaraldehyde and isomyristicin, tentatively identified based on their UV and HRMS data. In the literature, we found only two phytochemical studies of an organic extract from *M. depressa* stem bark, which had a different metabolic profile [30,31]. Our results agree with the report by Enriquez et al. [30], who identified α-asarone as the most abundant component in a hexane extract, which also included asaraldehyde, *trans*-isoelemicin and *trans*-isomyristicin. In the study by Jimenez Arellanes et al. [31], a chloroform extract contained four tetramethoxyl derivatives [1,2,3,4-tetramethoxy-5-(2-propenyl)-benzene, 2,3,4,5-tetramethoxybenzaldehyde, 2,3,4,5-tetramethoxycinnamaldehyde, 2,3,4,5-tetramethoxycinnamyl alcohol] and *trans*-isomyristicin. Such differences in composition could be attributed to season, phenological stage and geographical region where plants were collected, which can greatly influence chemical biosynthesis and bioactivity. For example, essential oils from *Perilla frutescens* collected from 11 areas in China differed in yields and chemical composition, which were associated with antioxidant and antifungal activities [42]. When total alkaloids and the annomontine and oxopurpureine content from roots and leaves of *Annona purpurea* were monitored over time, the alkaloid was high during the dry season and during flowering; the strongest antifungal activity was obtained from the root extracts during the last month of the dry season [43].

In our investigation, α-asarone (syn. *trans*-asarone) was identified as the principal compound responsible for the antifungal effect on *F. equiseti* and *F. oxysporum*. Its IC_50_ (236 and 482 µg/mL, respectively) and IC_95_ (269 and 526 µg/mL, respectively) were lower than those of the EE from *M. depressa* stem bark. An antifungal effect of α-asarone at 1000 mg/L has been reported for the phytopathogens *Phytophthora infestans* and *Pyricularia grisea* with growth inhibition (GI) of 85 and 53%, respectively [44], for *Botrytis cinerea*, *F. oxysporum* and *Phomopsis obscurans* (GI = 57.7, 43.6 and 41.5%, respectively) at 300 µM [45] and slight activity against the yeasts *Candida albicans*, *C. kruseii* and *C. parapsilasis* at 100 µg/mL [46]. It also has pesticidal properties as an antifeedant against *Helicovarpa zea*, *Helionthis virescens* and *Manduca sexta*; it is insecticidal against *Aedes aegypti* and *Lucila sericata*, and nematocidal against *Caenorhabditis elegans*, *Panagrellus redivivus* and *Nyppostrongylus brasiliensis* [46,47]. Interestingly, Jimenez Arellanes et al. [31] reported that 1,2,3,4-tetramethoxy-5-(2-propenyl)-benzene was the most abundant component in the chloroform extract (0.71% from dried stem bark) and the major phytogrowth inhibitory compound in *Amaranthus hychondriacus* (IC_50_ = 43 µg/mL) and *E. crusgalli* (IC_50_ = 43 µg/mL), and it had an antifungal effect on an undocumented strain of *F. oxysporum* (MIC: 250 µg/mL). In the present study, this compound was not detected from the stem extracts. However, it was abundant in the MDR-b fraction from *M. depressa* root bark, but it had no effect on the mycelial growth of *F. oxysporum* strain FCHJ, and *F. equiseti* strain FCHE was only moderately sensitive (75% MGI at 1000 µg/mL). Based on these results, the antifungal activity of *M. depressa* collected in Jahuactal is considered to be primarily due to the presence of α-asarone in the extract.

As shown by SEM, EE from *M. depressa* stem bark at 2000 µg/mL caused prominent morphological alterations of *F. oxysporum* and *F. equiseti*. Hyphae were malformed and contorted, and microconidia had collapsed. This effect is similar to the morphological changes in conidia and hyphae of the filamentous zoopathogenic fungus *Microsporum gyseum* after 4 d exposure to 100 mg/mL of the β-asarone fraction [48]; further cell death of *F. oxysporum* induced by a mixture of asarones (α, β, γ, 3.4:94.3:1%) at 500 µg/mL was observed using epifluorescence microscopy; the rapid cell death is correlated with greater production of reactive oxygen species [49]. Studies on the mechanism of action of β-asarone showed that it interferes with ergosterol synthesis, thus the ergosterol content is lowered in the plasma membrane of *Aspergillus niger* ATCC 16,888 [50], confirming that the effect against *F. oxysporum* might be related to the inhibition of ergosterol biosynthesis, as it is in *C. albicans* [51]. Hence, similar to its isomer β-asarone, α-asarone in the EE from *M. depressa* stem bark might inhibit the mycelial growth of *F. oxysporum* and *F. equiseti* by damaging the plasma membrane and causing cell death. More studies are needed to verify the site of action of asarones and other metabolites of *M. depressa* on fungal pathogens.

Another promising plant species for antifungal compounds in our study was *P. neesianum* (Piperaceae, syn. *Piper sempervirens, Arctottonia sempervirens*), a tree used in traditional medicine to treat snake bites and wounds [52,53]. The EE from *P. neesianum* leaves and its PNH-b fraction completely inhibited the growth of *F. equiseti* (MIC: 1000 µg/mL) and had the same IC_50_ and IC_95_ (462 and 866 µg/mL, respectively) as the MDT-b fraction. This report is the first of an antifungal effect of *P. neesianum* against *F. equiseti* and *F. oxysporum.* The dichloromethane extract from leaves of *P. neesianum* has been reported to have various biological activities as an antioxidant (IC_50_ = DPPH 0.071 mg/mL) [54], anti-tyrosinase (IC_50_ = 6.6 µg/mL [55] and anti-urease (IC_50_ = 12.9 µg/mL) [56]. Essential oil from *P. neesianum* leaves collected in the northern region of Guatemala contained bicyclogermacrene (28%), germacrene D (11.7%) and β-caryopyllene (7.5%) as major compounds among 19 detected in a gas chromatography with flame ionization detection- mass spectrometry analysis [52].

The EE from *P. cubana* (Primulaceae; syn. *Ardisia cubana*) roots was also active against both *Fusarium* pathogens, and the low polarity PCR-a fraction was fungicidal (MIC: 1000 µg/mL). These findings are the first report of a biological activity for extracts from *P. cubana*.

The EA from *C. jamaicensis* (Asteraceae) was the only EA that moderately inhibited the growth of *F. equiseti*, suggesting that it produces a highly polar antifungal metabolite(s). This species was documented to have leishmanicidal activity and to be useful for treating colds and stomach pain [57,58], but the present report is the first on its antifungal activity. Among 125 *Calea* species, only *C. urticifolia* has been tested against fungal pathogens, but it had no activity against *F. oxysporum* [25,59]. Acacetin, *O*-methylacacetin, jamaicolides A–D and prumichromene B have been identified in aerial parts of *C. jamaicensis* [58].

In summary, the present investigation revealed that *F. equiseti* FCHE and *F. oxysporum* FCHJ strains isolated from habanero pepper plants were sensitive to extracts from six native plant species, and the most effective were the EEs from *M. depressa*, *P. cubana* and *P. neesianum*, and advanced our knowledge about the phytochemicals in the roots of *M. depressa* from the Yucatán Peninsula. α-Asarone was identified as the principal antifungal component in the stem bark of *M. depressa*. Now, we need to determine the persistence of its antifungal effect and any toxicity to the environment and beneficial macro- and microorganisms in the soil as a pure compound and in the complex ethanolic extract mixture.

Our knowledge on the pesticidal potential of the native Mexican flora has also been enriched, and on the basis of our broad screening, we will isolate and identify the compounds in the active EEs from *P. cubana* and *P. neesianum* and the AE from *C. jamaicensis* that contribute to the antifungal activity. Subsequently, we expect to propagate the promising species to provide material for greenhouse and field experiments. Of course, the mechanism and sites of action of the identified metabolites in the fungus need to be determined, and the metabolites tested for safety against nontarget organisms. This research also opens opportunities for future studies on the conservation and sustainable use of our regional flora in the development of biorational products for the integrated management of *C. chinense* and other species of *Capsicum*.

## 4. Materials and Methods

### 4.1. Plant Materials

Plants were collected from six locations in the Yucatán Peninsula: (1) Jahuactal, Ejido Caobas, Othón Pompeyo Blanco (18°15′34″ N, 88°57′14″ W), (2) Kaxil Kiuic, Oxkutzcab (20°06′10.8″ N; 89°33′43.2″ W), (3) Punta Laguna, Valladolid (20°38′49.4″ N, 87°38′02.2″ W), (4) Xmaben, Hopelchén (19°15′42.92″ N, 89°21′45.91″ W), (5) Punta Pulticub, Othón P. Blanco (19°04′29.96″ N, 87°33′17.15″ W) and (6) Chacchoben Limones, Othón P. Blanco (19°01′44.31″ N, 88°08′00.38″ W) of the states of Yucatán and Quintana Roo (Table 1). Each plant was separated into leaves, stems and roots for separate extractions, and whole plants (WP) of some species were extracted. Plant materials were dried in a lamp stove at 55–60 °C for 5 d and crushed in a mill (model 1520, Pagani, Azcapotzalco, México) with blades and no. 5 mm mesh. A voucher specimen for each plant species was deposited in the Roger Orellana Herbarium of the Unidad de Recursos Naturales del Centro de Investigación Científica de Yucatán and identified by experts (Table 1).

### 4.2. Preparation of Plant Extracts

#### 4.2.1. Aqueous Extracts

The dried, ground plant material (1.5 g) was transferred to an Erlenmeyer flask, and 20 mL of boiling distilled water were added. After 15 min, the sample was filtered through filter paper (Whatman no. 1) and cotton to remove solid residues, then diluted with distilled water to 25 mL, to obtain an aqueous extract (AE) with a final concentration of 6% (*w*/*v*). Under aseptic conditions, the infusion was sterilized using a 0.22 μm Millipore filter (Merck-Millipore, Burlington, MA, USA), and frozen at −17.5 ± 0.5 °C until use [60].

#### 4.2.2. Ethanolic Extracts

The dried, ground plant material was immersed in ethanol (1.5% of the total volume) and extracted three times with ethanol by sonication at 20 kHz (Cole-Parmer, Chicago, IL, USA), at room temperature for 20 min each time. The solvent was filtered and eliminated under vacuum in a rotary evaporator (IKA model RV-10, Staufen, Germany) at 40 °C to obtain the ethanolic crude extract [24]. The EEs with the greatest activity in the antifungal assay described (Section 4.4) were partitioned with hexane–acetonitrile three times (2: 1, 1: 1, 1: 1 *v/v*) and solvents removed as described above. In this way, a hexane fraction (A), acetonitrile fraction (B) and methanol-soluble precipitate (C) of each EE were obtained.

### 4.3. Fungal Cultures

Phytopathogenic strains of *Fusarium equiseti* (FCHE, GenBank acc. MG020433) and *F. oxysporum* (FCHJ, GenBank acc. MG020428) were obtained from the fungal collection of the Phytopathology Laboratory, Tecnológico Nacional de México, Instituto Tecnológico de Conkal. These strains were isolated from stem and root lesions of habanero pepper plants [10]. The strains were maintained by transferring a mycelial disc (5 mm diameter) to (a) 20% glycerol (*v/v*) and frozen at −80 °C, (b) sterile distilled water and (c) commercial potato dextrose agar in slant tubes (PDA, BD, Bioxon, Edo. México) and stored at 4 °C in the dark.

### 4.4. Antifungal Microdilution Assay of Extracts

#### 4.4.1. Preparation of Conidial Suspension

*F. equiseti* and *F. oxysporum* strains were reactivated on PDA and incubated at 27 ± 2 °C, with 16 h light/8 h dark in a humidity chamber to induce sporulation. After 7 days, the surface of the culture was flooded with a sterile saline solution (5 mL), then gently scraped with a sterile brush to release conidia into the saline. The resulting conidial suspension was filtered through a double layer of sterile cheesecloth and adjusted to a final concentration of 1 × 10^5^ conidia/mL for both pathogens with sterile saline solution, using a hemocytometer [61].

#### 4.4.2. Bioassay with Aqueous Extracts

In the broth microdilution to determine the mycelial growth inhibition (MGI) of the *Fusarium* strains, 100 µL of each 6% AE were transferred to each microwell of a 96-well plate. As a negative control, 100 µL of the conidial suspension were used and as positive control, 5 µL of the fungicide Mirage CE 45 (prochloraz 450 g a.i./L) (Bayer CropScience, NC, USA). Finally, 100 µL of the conidial suspension were added for a final concentration of 3% *w*/*v* AE, 0.112% of prochloraz (*w*/*v*), 5 × 10^4^ conidia/mL of *Fusarium* strains. All tests were performed in triplicate and microdilution plates maintained at 27 ± 2 °C, and 16 h light/8 h dark. The MG was recorded at 96 h, visually determined with a microscope at 50× using the National Committee for Clinical Laboratory Standards with slight modifications, using a 0–4 scale, where 4 is full MG (0% MGI) and 0 the absence of MG (MGI =100%) [62,63]. Data were converted to a percentage of mycelial growth inhibition (MGI) using Abbott’s formula: [(% MG in the negative control − % MG in the treatment)/% MG in the negative control)] × 100 [62].

#### 4.4.3. Bioassay with Ethanolic Extracts

Each EE was dissolved in a mixture of dimethylsulfoxide (DMSO) (Sigma-Aldrich, St. Louis, MO, USA) with 0.5% Tween 20 to obtain a solution at 40 µg/µL EE. Then 10 µL of this EE solution were added to each microwell, containing 90 µL of RPMI liquid medium (Roswell Park Memorial Institute 1640). Mirage CE 45 (5 µL) was used as the positive control as described above; negative growth controls were RMPI (Merck Millipore Darmstadt, Germany), water (100 µL) and a blank (0.5% Tween 20 DMSO: RPMI 1:9, *v/v*). Each microwell then received 100 µL of the conidial suspension for a final EE concentration of 2000 µg/mL and 5% of DMSO with 0.5% Tween 20 (Merck Millipore Darmstadt, Germany) [24]. All tests were done three times, and the plates were incubated and assessed as described above.

#### 4.4.4. Minimum Inhibitory Concentration of Active EEs and Fractions

Serial dilutions of fractions A, B and C and active EE solutions (80 µg/µL), prepared as described above, were evaluated in a microdilution assay to determine the MIC [24]. The EEs were tested at final concentrations of 2000, 1000, 500 and 250 µg/mL. The fractions were evaluated at 1000, 500 and 250 µg/mL. The commercial α-asarone standard (Sigma-Aldrich, St. Louis, MO, USA) was tested at 500, 250 and 125 µg/mL. The same controls and incubation conditions were used as described above. All determinations were made with four replicates, three times. After incubation at 96 h, the MIC was determined as the lowest concentration of the extract at which no mycelial growth was observed in the well.

After 96 h of incubation, 10 µL from each well that had no growth were transferred to PDA and incubated at 27 ± 2 °C. After 72 h, the presence of growth was cataloged as fungicidal, the absence of growth as fungistatic [64].

### 4.5. Effect of Ethanolic Extracts on Hyphal Morphology of Fusarium Strains

The strains of *F. equiseti* and *F. oxysporum* were grown on PDA in Petri dishes for 7 d, then 5 mm disks were removed from the growing edge of the colony. The samples were fixed in 2.5% *v/v* glutaraldehyde (Merck Millipore Darmstadt, Germany) and 0.2 M sodium phosphate (Sigma-Aldrich, St. Louis, MO, USA) pH 7.2 for 48 h at 4 °C and washed twice with the phosphate buffer (1 h each time). The samples were dehydrated in an ethanol series (1 h each: 30, 50, 70, 85, 96 and 100%, 2 × absolute ethanol). The samples were dried with CO_2_ in a Sandri-795 critical point dryer (Tousimis Research Corp., Rockville, MD, USA), then attached to a sample holder using double-sided adhesive carbon tape and coated with gold for 10 min in an ionizing chamber (Dentom Vacuum-Desk II, Moorestown, NJ, USA). The samples were observed in a JSM 6360 SEM (Jeol, Tokyo, Japan) at 20 kV.

After the fungus was exposed for 96 h to 200 µL of EE from *M. depressa*, the mixture was filtered through a nylon membrane (nucleic acid blotting membrane Hybond N^+^ 0.45 µm) (GE Healthcare Bioscience, Amersham PI, Little Chalfont, UK), and the fungal samples were fixed as described above.

### 4.6. Chromatographic and Spectrometric Analyses

#### 4.6.1. Thin Layer Chromatography (TLC)

The active EEs and their fractions were analyzed by thin layer chromatography (TLC) using an aluminum support impregnated with 0.25 mm thick G-60 silica gel with fluorescent indicator F_254_ (Merck Millipore, Burlington, MA, USA). In parallel, the commercial standard α-asarone (Sigma-Aldrich, St. Louis, MO, USA) was applied to confirm its presence in *M. depressa* extracts. The plates were developed in three elution systems: hexane-acetone (8:2), CH_2_Cl_2_-AcOEt (9:1) and CH_2_Cl_2_-MeOH (85:15). After separation, the metabolites were visualized with ultraviolet light (UV_254_ and UV_365_) and phosphomolybdic acid (Sigma-Aldrich, St. Louis, MO, USA).

#### 4.6.2. LC-UV-HRMS

The active fractions (2 µL) from *M. depressa* stem bark (MDT-b) and root bark (MDR-b) were analyzed by liquid chromatography–ultraviolet–high-resolution mass spectrometry (LC-UV-HRMS) using a data-dependent acquisition protocol [65]. Chromatograms and mass spectra were obtained using an LC-MS (Agilent, Santa Clara, CA, USA) coupled to a Bruker Maxis HR-QTOF mass detector (Bruker Daltonics GmbH, Bremen, Germany) at 40 °C. A Zorbax SB-C8 column (Agilent, Santa Clara, CA, USA) was used (2.1 × 30 mm) with a mobile phase of a mixture of solvent A (water–acetonitrile 90:10 with 0.01% *v/v* trifluoroacetic acid and 1.3 mM ammonium formate) and solvent B (water–acetonitrile 10:90 with 0.01% *v/v* trifluoroacetic acid and 1.3 mM ammonium formate) and a flow rate of 300 µL/min. The gradient was set for a constant flow rate of 10% B to 100% B in 6 min, 100% B for 2 min, then 10% B for 2 min. Mass spectra (150 to 2000 *m/z*) were acquired in positive mode. The components detected were compared with the MEDINA database of microbial metabolites and the Chapman & Hall Dictionary of Natural Products (v25.1, CRC Press, Boca Raton, FL, USA).

### 4.7. Statistical Analyses

For the % MGI data, a one-way analysis of variance was performed with prior transformation of the original data using the formula: *y* = arsin [sqrt (*y*/100)]. The treatment means were compared using Tukey’s multiple range test (*p* = 0.05). Variance analyses were performed using SAS ver. 9.4 for Windows (SAS Institute, Cary, NC, USA). IC_50_ and IC_95_ values with 95% confidence intervals were calculated for EEs and effective fractions using a probit analysis.

## Figures and Tables

**Table 1:** Plants collected from the Yucatán Peninsula to screen for activity against *F. equiseti* strain FHCE and *F. oxysporum* strain FCHJ.

Species	Local Name ^a^	Family	Site	Voucher	Plant Parts Used
*Alseis yucatanensis* Standl.	ja’as che’	Rubiaceae	Kiuic	JLT-3179	L
*Alvaradoa amorphoides* Liebm.	bel siinik che’	Simaroubaceae	Jahuactal	GC-8236	L, S, R
*Annona primigenia* Standl. & Steyerm		Annonaceae	Jahuactal	GC-8057	L, SB
*Bakeridesia notolophium* (A. Gray) Hochr.		Malvaceae	Punta Pulticub	RD-s/n	L, S
*Bravaisia berlandieriana* (Nees) T.F.Daniel	Juluub	Acanthaceae	Punta Laguna	GC-8168	L, S, R
*Byrsonima bucidifolia* Standl.		Malpighiaceae	Jahuactal	GC-8087	L, S, R
*Calea jamaicensis* (L.) L.	tu’ xikin	Asteraceae	Jahuactal	GC-8084	WP
*Cameraria latifolia* L.	cheechen blanco	Apocynaceae	Jahuactal	JLT-1165	L, SB, R
*Chrysophyllum mexicanum* Brandegee ex Standl.	chi’kéej	Sapotaceae	Jahuactal	GC-8082	L, S, R
*Coccoloba* sp.		Polygonaceae	Xmaben	GC-8258	L, S
*Croton arboreus* Millsp.	pak che’	Euphorbiaceae	Jahuactal	JLT-1132	L, S, R
*Croton itzaeus* Lundell		Euphorbiaceae	Jahuactal	JLT-1138	L, SB, RB
*Croton* sp.		Euphorbiaceae	Xmaben	GC-8262	WP
*Cupania* sp.		Sapindaceae	Chacchoben Limones	GC-8009	L, S
*Diospyros* sp.		Ebenaceae	Punta Laguna	GC-8147	L
*Erythroxylum confusum* Britton		Erythroxylaceae	Jahuactal	JLT-1143	L, S, R
*Erythroxylum rotundifolium* Lunan	baak soots’	Erythroxylaceae	Jahuactal	GC-8179	L, S
*Erythroxylum* sp.		Erythroxylaceae	Punta Laguna	GC-8137	L
*Eugenia* sp.		Myrtaceae	Punta Laguna	GC-8127	L, S, R
*Euphorbia armourii* Millsp.	kabal chakaj	Euphorbiaceae	Kaxil Kiuic	JLT-3182	WP
*Guettarda combsii* Urb.		Rubiaceae	Jahuactal	GC-8047	L, SB, RB
*Helicteres baruensis* Jacq.	Sutup	Malvaceae	Kaxil Kiuic	GC-8127	L, S, R
*Heteropterys laurifolia* (L.) A. Juss.	chilillo aak’	Malpighiaceae	Jahuactal	GC-8035	L, SB, R
*Hybanthus yucatanensis* Millsp.		Violaceae	Punta Laguna	GC-8158	L, S
*Ipomoea clavata* (G. Don) Ooststr. ex J.F.Macbr.	ulu’um ja’	Convolvulaceae	Kaxil Kiuic	JLT-3181	WP
*Karwinskia humboldtiana* (Willd. ex Roem. & Schult.) Zucc.	I u’um che’	Rhamnaceae	Kaxil Kiuic	JLT-3188	L
*Licaria* sp.		Lauraceae	Jahuactal	GC-8037	L, SB, RB
*Macroscepis diademata* (Ker Gawl.) W.D. Stevens	aak’tóom paap	Apocynaceae	Kaxil Kiuic	JLT-3187	L, SB
*Malpighia glabra* L.		Malpighiaceae	Punta Laguna	GC-8144	L, S, R
*Morella cerifera* (L.) Small.		Myricaceae	Jahuactal	JLT-1137	L, S, RB
*Mosannona depressa* (Ball.) Chatrou	sak éelemuy	Annonaceae	Jahuactal	GC-8085	L, SB, RB
*Parathesis cubana* (A. DC.) Molinet & M.Gómez		Primulaceae	Jahuactal	JLT-1133	L, SB, RB
*Paullinia* sp.		Sapindaceae	Punta Laguna	GC-8106	L, R
*Piper neesianum* C.DC.		Piperaceae	Jahuactal	GC-8080	L, S, R
*Psychotria* sp.		Rubiaceae	Jahuactal	GC-8086	WP
*Randia aculeata* L.	kat ku’uk	Rubiaceae	Punta Laguna	GC-8156	L, S, R
*Serjania caracasana* (Jacq.) Willd		Sapindaceae	Punta Laguna	GC-8114	L, S, R
*Simarouba glauca* DC.		Simaroubaceae	Jahuactal	GC-8081	L, SB, RB
*Stemmadenia donnell-smithii* (Rose) Woodson		Apocynaceae	Jahuactal	GC-8056	L, SB
*Turnera aromatica* Arbo		Passifloraceae	Jahuactal	GC-8081	WP

^a^ [27]; SB: stem bark; RB: root bark; L: leaves; S: stem; R: root; WP: whole plant.

**Table 2:** Inhibition of mycelial growth of *Fusarium equiseti* strain FCHE and *F. oxysporum* strain FCHJ by active plant extracts from native species of the Yucatán Peninsula in microdilution assay.

Extract Concentration	Plant Species	Mycelial Growth Inhibition (%)									
		*Fusarium equiseti*					*Fusarium oxysporum*				
		L	S	R	WP		L	S	R	WP	
Ethanolic	*Mosannona depressa*	0 ^c^	100 ^a^	100 ^a^	ne		0 ^b^	100 ^a^	100 ^a^	ne	
2000 µg/mL	*Parathesis cubana*	0 ^c^	0 ^b^	100 ^a^	ne		0 ^b^	0 ^b^	100 ^a^	ne	
	*Piper neesianum*	100 ^a^	0 ^b^	0 ^c^	ne		75 ^a^	0 ^b^	0 ^b^	ne	
Aqueous	*Cameraria latifolia*	0 ^c^	0 ^b^	25 ^b^	ne		0 ^b^	0 ^b^	0 ^b^	ne	
3% *w*/*v*	*Calea jamaicensis*	ne	ne	ne	75		ne	ne	ne	0	
	*Heteropterys laurifolia*	25 ^b^	0 ^b^	0	ne		0 ^b^	0 ^b^	0 ^b^	ne	
Negative C	RPMI					0 ^b^					0 ^b^
	blank					0 ^b^					0 ^b^
Positive C	Prochloraz 0.11%					100 ^a^					100 ^a^

C: control; L: leaves; S: stem, R: root; WP: whole plant; RPMI: Roswell Park Memorial Institute medium; ne: not evaluated; blank: dimethyl sulfoxide with 0.5% Tween 20. ^a, b, c^: means with different letters within columns differ significantly (Tukey’s test, *p* < 0.05). Extracts from *M. depressa* were from bark of stems and roots.

**Table 3:** Minimum inhibitory concentration (MIC) of extracts and fractions from *Mosannona depressa* (bark of stems and roots), *P. cubana* (roots), *P. neesianum* (leaves) and α-asarone against *Fusarium equiseti* strain FCHE and *F. oxysporum* strain FCHJ.

Extract/ Fraction	Solvent	*Fusarium equiseti*					*Fusarium oxysporum*				
		Concentration of Extracts (µg/mL)									
		2000	1000	500	250	MIC	2000	1000	500	250	MIC
MDT	E	100 ^a^	100 ^a^	75 ^c^	0 ^c^	1000++	100 ^a^	75 ^b^	0 ^f^	0 ^b^	2000+
MDT-a	H	ne	100 ^a^	0 ^e^	0 ^c^	1000++	ne	100 ^a^	0 ^f^	0 ^b^	1000+
MDT-b	A	ne	100 ^a^	83 ^b^	0 ^c^	1000++	ne	100 ^a^	75 ^b^	0 ^b^	1000+
MDT-c	P	ne	75 ^c^	50 ^d^	0 ^c^	>1000	ne	75 ^b^	50 ^d^	0 ^b^	>1000
MDR	E	100 ^a^	83 ^b^	0 ^e^	0 ^c^	2000++	100 ^a^	75 ^b^	0 ^f^	0 ^b^	2000+
MDR-a	H	ne	0 ^d^	0 ^e^	0 ^c^	>1000	ne	0 ^d^	0 ^f^	0 ^b^	>1000
MDR-b	A	ne	75 ^c^	0 ^e^	0 ^c^	>1000	ne	0 ^d^	0 ^f^	0 ^b^	>1000
MDR-c	P	ne	83 ^b^	50 ^d^	0 ^c^	>1000	ne	75 ^b^	50 ^d^	0 ^b^	>1000
PCR	E	100 ^a^	100 ^a^	0 ^e^	0 ^c^	1000++	100 ^a^	58 ^c^	25 ^e^	0 ^b^	2000+
PCR-a	H	ne	100 ^a^	0 ^e^	0 ^c^	1000++	ne	100 ^a^	0 ^f^	0 ^b^	1000++
PCR-b	A	ne	100 ^a^	0 ^e^	0 ^c^	1000++	ne	0 ^d^	0 ^f^	0 ^b^	>1000
PCR-c	P	ne	0 ^d^	0 ^e^	0 ^c^	>1000	ne	0 ^d^	0 ^f^	0 ^b^	>1000
PNH	E	100 ^a^	100 ^a^	0 ^e^	0 ^c^	1000+	75 ^b^	ne	ne	ne	2000+
PNH-a	H	ne	0 ^d^	0 ^e^	0 ^c^	>1000	ne	ne	ne	ne	
PNH-b	A	ne	100 ^a^	83 ^b^	0 ^c^	1000++	ne	ne	ne	ne	
PNH-c	P	ne	100 ^a^	0 ^e^	0 ^c^	1000+	ne	ne	ne	ne	
α-Asarone	CS		ne	100^a^	75 ^b^	500++	ne	ne	66 ^c^	0 ^b^	>500
NC		0 ^b^	0 ^d^	0 ^e^	0 ^c^		0 c	0 ^d^	0 ^f^	0 ^b^	
PC		100 ^a^	100 ^a^	100 ^a^	100 ^a^		100 ^a^	100 ^a^	100 ^a^	100 ^a^	

MDT: *Mosannona depressa* (stem bark); MDR: *M. depressa* (root bark); PCR: *Parathesis cubana* (root); PNH: *Piper neesianum* (leaves); -a, -b, -c: nomenclature of fractions related with the solvent used; NC: negative control (conidial suspension/RPMI: Roswell Park Memorial Institute medium); PC: positive control (prochloraz 0.11%); E: ethanol; H: hexane; A: acetonitrile; P: precipitate; CS: commercial standard; ne: not evaluated; (++): fungicidal; (+): fungistatic. ^a, b, c, d^: Means with different letters within columns differ significantly (Tukey’s test, *p* < 0.05).

**Table 4:** IC_50_ and IC_95_ of active extracts and fractions from *Mosannona depressa*, *Parathesis cubana*, *Piper neesianum* and of the commercial standard α-asarone against mycelial growth of *Fusarium equiseti* strain FCHE and *F. oxysporum* strain FCHJ.

Source	Extract/Fraction	*Fusarium equiseti*		*Fusarium oxysporum*	
		IC_50_ (CI)	IC_95_ (CI)	IC_50_ (CI)	IC_95_ (CI)
*M. depressa*	MDT	468 (455–477)	545 (534–561)	944 (889–965)	1079 (1051–1156)
	MDT-b	462 (412–476)	526 (515–562)	472 (432–483)	539 (524–596)
α-asarone	CS	236 (216–244)	269 (259–289)	482 (459–494)	526 (521–582)
*P. cubana*	PCR	788 (545–984)	866 (638–1063)	876 (836–920)	1494 (1407–1602)
*P. neesianum*	PNH	788 (545–984)	866 (638–1063)	ne	ne
	PNH-b	462 (412–476)	526 (515–562)	ne	ne

CI: confidence interval; CS: commercial standard; MDT: *Mosannona depressa* (stem bark); PCR: *Parathesis cubana* (root); PNH: *Piper neesianum* (leaves); b: acetonitrile fraction; ne: not evaluated.

**Table 5:** Metabolites identified from acetonitrile fraction of *Mosannona depressa* stem bark (MDT-b) by liquid chromatography–ultraviolet–high-resolution mass spectrometry (LC-UV-HRMS).

Peak	Retention Time (min)	[M + H]^+^	MW	Molecular Formula	Compound
1	2.23	225.1120	224.1120	C_12_H_16_O_4_	Not identified
2	2.55	197.0808	196.0735	C_10_H_12_O_4_	Asaraldehyde
3	4.27	209.1172	208.1099	C_12_H_16_O_3_	α-Asarone
4	4.81	221.1170	220.1097	C_13_H_16_O_3_	Not identified
5	4.89	193.0857	192.0784	C_11_H_12_O_3_	Isomyristicin

MW: molecular weight.

**Table 6:** Compounds identified in the acetonitrile fraction of the ethanolic extract of *Mosannona depressa* root bark (MDR-b) using LC-UV-HRMS.

Peak	Retention Time (min)	[M + H]^+^	MW	Molecular Formula	Compound
1	4.25	209.1172	208.1094	C_12_H_16_O_3_	α-Asarone
2	4.37	239.1278	238.1205	C_13_H_18_O_4_	1,2,3,4-Tetramethoxy-5- (2-propenyl) benzene

MW: molecular weight.

## Supplementary Materials

The following are available online at https://www.mdpi.com/2076-0817/9/10/827/s1, Table S1: Inhibition of mycelial growth of *Fusarium equiseti* strain FCHE and *F. oxysporum* strain FCHJ by plant extracts from 40 native species of the Yucatán Peninsula in microdilution assay.
