# Supplementary material for: Extracts from Six Native Plants of the Yucatán Peninsula Hinder Mycelial Growth of Fusarium equiseti and F. oxysporum, Pathogens of Capsicum chinense
Source: Pathogens. 2020 Oct 10;9(10):827. doi: 10.3390/pathogens9100827 (PMC7601340; doi:10.3390/pathogens9100827)
Supplement: Supplementary file 1 [file pathogens-09-00827-s001.pdf]

Table S1. Inhibition of mycelial growth of *Fusarium equiseti* strain FCHE and *F. oxysporum* strain FCHJ by plant extracts from 40 native species of the Yucatán Peninsula in microdilution assay.

Plant species	Mycelial Growth Inhibition (%)															
	Ethanolic extract (2,000 μg/mL)								Aqueous extract (3%, p/v)							
	Fusarium equiseti				*Fusarium oxysporum*				*Fusarium equiseti*				*Fusarium oxysporum*			
	L	S	R	WP	L	S	R	WP	L	S	R	WP	L	S	R	WP
Alseis yucatanensis	0	ne	ne	ne	0	ne	ne	ne	0	ne	ne	ne	0	ne	ne	ne
Alvaradoa amorphoides	0	0	0	ne	0	0	0	ne	0	0	0	ne	0	0	0	ne
Annona primigenia	0	0	ne	ne	0	0	ne	ne	0	0	ne	ne	0	0	ne	ne
Bakeridesia sp.	0	0	ne	ne	0	0	ne	ne	0	0	ne	ne	0	0	ne	ne
Bravaisia berlandieriana	0	0	0	ne	0	0	0	ne	0	0	0	ne	0	0	0	ne
Byrsonima bucidifolia	0	0	0	ne	0	0	0	ne	0	0	0	ne	0	0	0	ne
Calea jamaicensis	ne	ne	ne	0	ne	ne	ne	0	ne	ne	ne	75	ne	ne	ne	0
Chrysophyllum mexicanum	0	0	0	0	0	0	0	0	0	0	0	0	0	0	0	0
Cameraria latifolia	0	0	0	0	0	0	0	0	0	0	25	ne	0	0	0	ne
Coccoloba sp.	0	0	0	ne	0	0	0	ne	0	0	0	ne	0	0	0	ne
Croton arboreus	0	0	0	0	0	0	0	0	0	0	0	0	0	0	0	0
Croton itzaeus	0	0	0	0	0	0	0	0	0	0	0	0	0	0	0	0
Croton sp.	ne	ne	ne	0	ne	ne	ne	0	ne	ne	ne	0	ne	ne	ne	0
Cupania sp.	0	0	ne	ne	0	0	ne	ne	0	0	ne	ne	0	0	ne	ne
Diospyros sp.	0	ne	ne	ne	0	ne	ne	ne	0	ne	ne	ne	0	ne	ne	ne
Erythroxylum confusum	0	0	0	ne	0	0	0	0	0	0	0	0	0	0	0	0
Erythroxylum rotundifolium	0	0	ne	ne	0	0	ne	ne	0	0	ne	ne	0	0	ne	ne
Erythroxylum sp.	0	ne	ne	ne	0	ne	ne	ne	0	ne	ne	ne	0	ne	ne	ne
Eugenia sp.	0	0	0	ne	0	0	0	ne	0	0	0	ne	0	0	0	ne
Euphorbia armourii	ne	ne	ne	0	ne	ne	ne	0	ne	ne	ne	0	ne	ne	ne	0
Guettarda combsii	0	0	0	ne	0	0	0	ne	0	0	0	ne	0	0	0	ne
Helicteres baruensis	0	0	0	ne	0	0	0	ne	0	0	0	ne	0	0	0	ne
Heteropterys laurifolia	0	0	0	ne	0	0	0	ne	25	0	0	ne	0	0	0	ne
Hybanthus yucatanensis	0	0	ne	ne	0	0	ne	ne	0	0	ne	ne	0	0	ne	ne
Ipomoea clavata	ne	ne	ne	0	ne	ne	ne	0	ne	ne	ne	0	ne	ne	ne	0

Karwinskia humboldtiana	0	ne	ne	ne		0	ne	ne	ne		0	ne	ne	ne		0	ne	ne	ne	
Licaria sp.	0	0	0	ne		0	0	0	ne		0	0	0	ne		0	0	0	ne	
Macroscepis diademata	0	0	ne	ne		0	0	ne	ne		0	0	ne	ne		0	0	ne	ne	
Malpighia glabra	0	0	0	ne		0	0	0	ne		0	0	0	ne		0	0	0	ne	
Morella cerifera	0	0	0	ne		0	0	0	ne		0	0	0	ne		0	0	0	ne	
Mosannona depressa	0	100	100	ne		0	100	100	ne		0	0	0	ne		0	0	0	ne	
Parathesis cubana	0	0	100	ne		0	0	100	ne		0	0	0	ne		0	0	0	ne	
Paullinia sp.	0	ne	0	ne		0	ne	0	ne		0	ne	0	ne		0	ne	0	ne	
Piper neesianum	100	0	0	ne		75	0	0	ne		0	0	0	ne		0	0	0	ne	
Psychotria sp.	0	0	0	ne		0	0	0	ne		0	0	0	ne		0	0	0	ne	
Randia aculeata	0	0	0	ne		0	0	0	ne		0	0	0	ne		0	0	0	ne	
Serjania caracasana	0	0	0	ne		0	0	0	ne		0	0	0	ne		0	0	0	ne	
Simarouba glauca	0	0	0	ne		0	0	0	ne		0	0	0	ne		0	0	0	ne	
Stemmadenia donnell-smithii	0	0	ne	ne		0	0	ne	ne		0	0	ne	ne		0	0	ne	ne	
Turnera aromatica	ne	ne	ne	0		ne	ne	ne	0		ne	ne	ne	0		ne	ne	ne	0	
RPMI					0					0					ne					ne
blank					0					0					0					0
Prochloraz 0.11%					100					100					100					100

C: control; L: leaves; S: stem, R: root; WP: whole plant; ne: not evaluated; RPMI: Roswell Park Memorial Institute medium; blank: dimethyl sulfoxide with 0.5% Tween 20. Extracts from *M. depressa* were from barks of stems and roots.
